# Pre-clinical Investigation of Rett Syndrome Using Human Stem Cell-Based Disease Models

**DOI:** 10.3389/fnins.2021.698812

**Published:** 2021-08-25

**Authors:** Florencia D. Haase, Bronte Coorey, Lisa Riley, Laurence C. Cantrill, Patrick P. L. Tam, Wendy A. Gold

**Affiliations:** ^1^Faculty of Medicine and Health, The University of Sydney, Sydney, NSW, Australia; ^2^Kids Neuroscience Centre, Kids Research, Children’s Hospital at Westmead, Westmead, NSW, Australia; ^3^Molecular Neurobiology Research Laboratory, Kids Research, Children’s Hospital at Westmead, and Children’s Medical Research Institute, Westmead, NSW, Australia; ^4^Rare Diseases Functional Genomics Laboratory, Kids Research, Children’s Hospital at Westmead, and Children’s Medical Research Institute, Westmead, NSW, Australia; ^5^Kids Research, Children’s Hospital at Westmead, Westmead, NSW, Australia; ^6^Embryology Research Unit, Children’s Medical Research Institute, The University of Sydney, Sydney, NSW, Australia; ^7^School of Medical Sciences, Faculty of Medicine and Health, The University of Sydney, Sydney, NSW, Australia

**Keywords:** Rett syndrome, neurodevelopmental disorders, iPSCs (induced pluripotent stem cells), brain organoids, disease modeling

## Abstract

Rett syndrome (RTT) is an X-linked neurodevelopmental disorder, mostly caused by mutations in *MECP2*. The disorder mainly affects girls and it is associated with severe cognitive and physical disabilities. Modeling RTT in neural and glial cell cultures and brain organoids derived from patient- or mutation-specific human induced pluripotent stem cells (iPSCs) has advanced our understanding of the pathogenesis of RTT, such as disease-causing mechanisms, disease progression, and cellular and molecular pathology enabling the identification of actionable therapeutic targets. Brain organoid models that recapitulate much of the tissue architecture and the complexity of cell types in the developing brain, offer further unprecedented opportunity for elucidating human neural development, without resorting to conventional animal models and the limited resource of human neural tissues. This review focuses on the new knowledge of RTT that has been gleaned from the iPSC-based models as well as limitations of the models and strategies to refine organoid technology in the quest for clinically relevant disease models for RTT and the broader spectrum of neurodevelopmental disorders.

## Introduction

**FIGURE 1:**
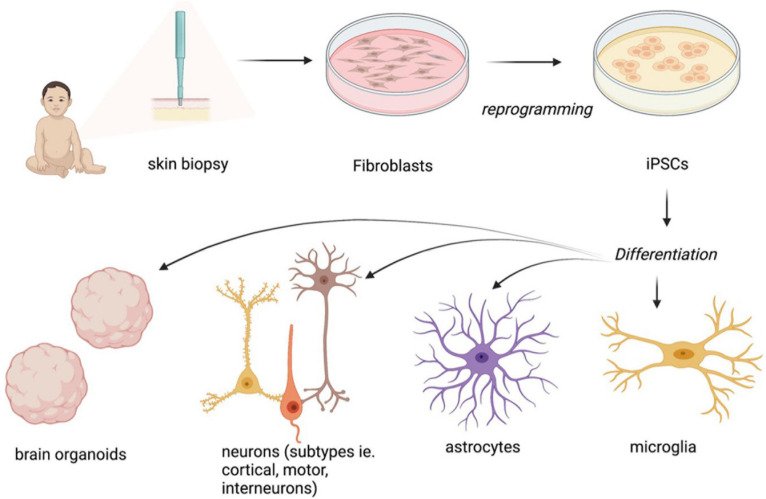
Normal MeCP2 function as a transcriptional repressor and activator and consequences of abnormal MeCP2 function in the brain at a cellular level. “Created with BioRender.com.”

Since the discovery that mutations in the MECP2 gene were the underlying cause of RTT, a great amount of work has been undertaken to understand the molecular function of MeCP2 (Amir et al., 1999; Figure 1). MeCP2 was initially identified as a transcriptional repressor located in the nucleus that bound to methylated DNA (Lewis et al., 1992). However, subsequently, it was described as a pluripotent transcriptional regulator, with the capacity to activate or repress target genes depending on the molecular context (Chahrour et al., 2008). More recently, the roles of MeCP2 have expanded to include alternative splicing, chromatin remodeling and miRNA processing (as reviewed in Tillotson and Bird, 2020). Patients with RTT, generally, present reduced brain volume associated with an abnormal morphology of neurons, including small and densely packed structures (Armstrong et al., 1995). It has also been observed that the dendrites of pyramidal neurons are significantly shorter than in wild type brains. Observations using MRI data have also shown reductions in the volume of the parietal and temporal lobes in RTT patients with conservation of the occipital cortex (Carter et al., 2008).

From a cellular point of view, it is known that MeCP2 loss affects different cell types in the brain. Region specific knockouts in mouse brains have shown several different phenotypes; for instance, removal of MeCP2 from dopaminergic and noradrenergic neurons induced motor incoordination (Samaco et al., 2009) and removal from the brainstem and spinal cord led to abnormal heart rate and breathing patters (Huang et al., 2016). Loss of MeCP2 in inhibitory neurons has also shown a prominent reproducibility of severe forms of RTT phenotypes, emphasizing the importance of E/I balance to MeCP2-mediated functions (Chao et al., 2010).

## Clinical Features of Rett Syndrome

Rett syndrome (RTT) is a rare neurodevelopmental disorder that manifests between 6 and 18 months of age after a period of apparently normal prenatal and postnatal development. Symptoms vary amongst individuals and include loss of fine and gross motor skills, abnormal social behavior, growth retardation, seizures, breathing dysregulation and stereotypic hand wringing movements (Neul et al., 2010). RTT has a worldwide prevalence of 1 in 10,000 individuals and predominantly affects females.

The majority of RTT cases are caused by *de novo* mutations in the X-linked methyl-CpG-binding protein 2 (*MECP2*) gene, which plays a critical role in normal brain development, specifically in the maturation of the central nervous system (CNS) and synapse development and function (Liyanage and Rastegar, 2014).

Substantial phenotypic variability is observed in girls with RTT, which is attributed to the type of mutation (missense, nonsense, etc.) and its location on *MECP2* as well as X chromosome inactivation (XCI). As the mutations observed in RTT girls are predominantly hemizygous, XCI renders a mosaic expression of the variant with some cell populations expressing the mutant *MECP2* allele and others, the wild type allele. While these two populations are normally present in nearly equal (1:1) proportion, the X inactivation pattern can be skewed, favoring the expression of one cell population over the other. In contrast, RTT males present with a homogenous population of mutant cells and are thus more severely affected.

Although *MECP2* is ubiquitously expressed, its expression is particularly high in post-mitotic neurons in the brain. Mutations that result in the dysregulation of *MECP2* cause a range of abnormalities at the anatomical, cellular, and molecular levels that is well characterized. There is an apparent reduction in brain volume in both humans and mouse models correlating with the severity of the phenotype (Carter et al., 2008), however, this reduction in brain size is not due to gross anatomical defects, but rather to small and densely packed neurons of reduced dendritic branching, soma size, spine density and synapse number (Belichenko et al., 2009).

Currently, treatment for RTT is purely symptomatic and requires a multidisciplinary approach for medical management. Furthermore, despite almost 60 observational and interventional clinical trials being conducted, it is still unknown whether all symptoms can be reversed. One of the most significant contributions to the field of RTT has been the phenotypic reversal of *Mecp2*-deficient transgenic mice to wild type upon the reinstatement of *Mecp2* expression, revealing the potential of gene therapy as a treatment (Guy et al., 2007). However, none of the recent gene transfer therapy studies in RTT mouse models have been able to entirely correct all RTT-associated symptoms (Gadalla et al., 2013, 2017; Garg et al., 2013; Sinnett et al., 2017; Tillotson et al., 2017; Luoni et al., 2020).

## Modeling Rett Syndrome Using iPSCs

The modeling of neurodevelopmental disorders is hampered by the inaccessibility and the limited source of live human brain tissue. Post-mortem tissue and immortalized cell lines have been adopted as the standard models for investigating the pathology of RTT predominately at the end-stage of the disease. Genetic mouse models of RTT that replicate salient features of the human neuropathology and cognitive deficits has further allowed the study of the pathophysiology and the natural history of disease progression. However, the inter-species differences in brain development and physiological function, and the inherent differentiation in cell composition between humans and mice at fetal and adult stages (Velasco et al., 2019; Eze et al., 2021) raise reservations about the fidelity of the animal study in modeling human neurodevelopmental disorders. Therefore, understanding of the disease causing mechanisms and pathophysiology of RTT with a view to developing effective therapies requires development of humanized disease models, which could be accomplished by using human pluripotent stem cells to generate cellular models and brain organoids.

**FIGURE 2:**
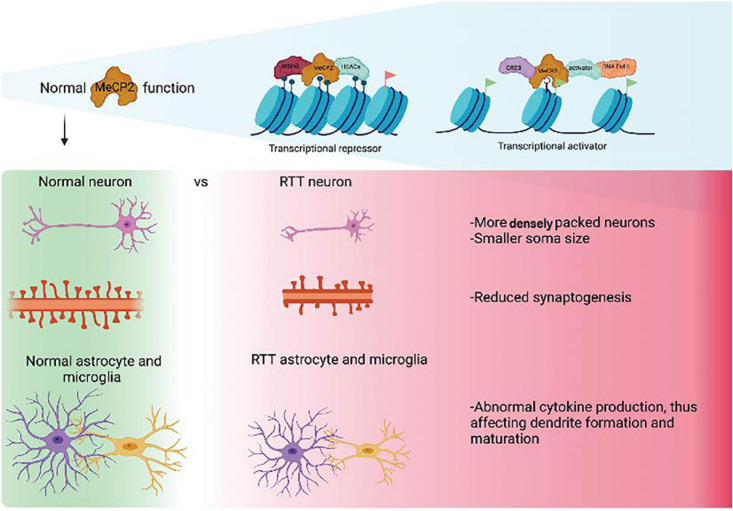
Overview of different methodologies employed in the articles used in this review to model RTT using iPSCs. “Created with BioRender.com.”

The introduction of human embryonic stem cells (hESCs) and induced pluripotent stem cells (iPSCs) to the discipline of precision medicine and advanced therapeutics has revolutionized the pre-clinical research of neurodevelopmental disorders (NDDs) such as RTT. hESCs are derived from blastocyst-stage human embryos whereas iPSCs are derived from somatic cells such as fibroblasts, blood and exfoliated urinary epithelial cells by cell reprogramming through transgenesis or foot-print free genetic modulation (Martinez-Fernandez et al., 2010; Yamanaka, 2012; Hsu et al., 2020). Both types of stem cells can be subjected to genetic modification to generate specific disease variants for modeling NDDs, where mutation- or patient- specific stem cells cannot be obtained. Stem cells have been used to create cellular models for a variety of cell types such as cortical and ventral neurons and glial cells in a conventional tissue culture setting (Takahashi and Yamanaka, 2006; Marchetto et al., 2010), as well as three-dimensional (3-D) complex tissue spheroids, organoids and assembloids that recapitulate different brain parts (Lancaster et al., 2013; Marton and Paşca, 2020; Figure 2). In brief, spheroids are simple clusters of cells that do not require scaffolding to form three dimensional structures and hence are not as advanced as organoids, which in contrast are more complex clusters of organ specific cells such as different types of neural cells; assembloids are structures formed by the assembly of different tissue specific organoids.

The use of iPSCs has been particularly useful as a complementary approach to existing disease models, such as murine and post-mortem tissue, as they offer a more human-centered approach in which to test therapies given the inaccessibility to patient brain tissue. Encouragingly, disease modeling has been achieved for neurodegenerative disorders such as Alzheimer’s, Parkinson’s, and Huntington’s disease, extending recently to neuropsychiatric, monogenetic, and chromosomal aberration disorders.

### Resource of MECP2-Mutant iPSCs

There are over 500 pathogenic mutations in *MECP2*, with the majority lying in exons 3 and 4 in the methyl binding domain (MBD), transcriptional repressor domain (TRD) and the C-terminal functional domain (CTD) regions, disrupting the normal function of the MeCP2 protein (Wan et al., 1999; Gold et al., 2018). Several iPSC lines harboring a range of *MECP2* mutations, including point mutations, frameshift mutations, deletions, and nonsense mutations have been generated and used in RTT studies (Supplementary Table 1).

All RTT studies using iPSC models have used iPSCs reprogrammed from patient-derived fibroblast cells (Table 1). As RTT manifests mostly in girls, the majority of studies to date have used iPSC lines from female patients, with the exception of five studies that used male patient-specific iPSCs (Tang et al., 2016; Zhang et al., 2016; de Souza et al., 2017; Yoo et al., 2017; Kim et al., 2019; Supplementary Table 1). To take into consideration the impact of XCI and genetic background of individuals, female lines are usually compared to their isogenic controls, which are genetically corrected iPSCs from the same patient (Kim et al., 2011; Cheung et al., 2012; Andoh-Noda et al., 2015; Djuric et al., 2015; Chin et al., 2016; Landucci et al., 2018; Nguyen et al., 2018). In studies where male lines are used, the control cell lines have been derived from the unaffected father, acting as a surrogate isogenic control (Tang et al., 2016; de Souza et al., 2017; Yoo et al., 2017; Kim et al., 2019).

**TABLE 1:** XCI status of different cell lines used.

**Group**	**Fibroblasts reprogrammed**	**XCI status of iPSC clones**
Cheung et al., 2011	MECP2: Δ3–4 (g.61340_67032delinsAGTTGTGCCAC and g.67072_67200del) – female, GM11270 (R306C) – female, GM17880 (T158M) – female	Retained Xi
Ananiev et al., 2011	MECP2: GM17880 (T158M) – female, GM07982 (V247*) – female, GM11270 (R306C) – female, RS0502 (R294*) – female	Retained Xi
Amenduni et al., 2011	CDKL5 (Q347*) – female CDKL5 (T288I) – male	Retained Xi
Pomp et al., 2011	MECP2: GM11272 (1155del32) – female, GM17880 (T158M) – female	Retained Xi
Marchetto et al., 2010	MECP2: GM11270 (R306C) – female, GM11272 (1155del32) – female, GM16548 (Q244*) – female, GM17880 (T158M) – female	Expressed Xi
Kim et al., 2011	MECP2: GM07982 (705delG) – female, GM11270 (R306C) – female, GM16548 (Q244*) – female, GM17567 (X487*) – female, GM17880 (T158M) -female	Expressed Xi

### Modeling RTT Neuronal Cells

Rett syndrome is an archetypical genetic disease and was one of the first pediatric neurological disorders to be studied using stem cell-based models (Xie and Tang, 2016). The phenotypes of neuronal cells of mice and post-mortem human brains include smaller soma size and reduced dendritic branching, spine density and morphology, as well as axonal bouton arborization and synapse formation (Belichenko et al., 2009; Marchetto et al., 2010; Baj et al., 2014; Figure 1). These anatomical defects are accompanied by an imbalance of excitatory/inhibitory (E/I) activity, with more excitatory and less inhibitory synaptic activity in the hippocampus and cortex (Nelson et al., 2006; Chao et al., 2010). iPSCs from RTT patient fibroblasts have been successfully differentiated into neuronal progenitor cells (NPCs), neurons and glia and the modeling has recapitulated some of the cellular and physiological phenotypes of these cell types, including morphological and electrophysiological defects.

The distinct phenotype of post-mortem and murine RTT neurons, characterized by a smaller soma size and reduced dendritic branching, spine density and axonal arborization, is well documented (Belichenko et al., 2009; Marchetto et al., 2010; Baj et al., 2014). This robust phenotype has been recapitulated in a number of patient-derived iPSCs harboring pathogenetic mutations in *MECP2*. Several studies have demonstrated that neurons differentiated from RTT-iPSCs in 2D cultures have a distinctly smaller soma size compared to that of controls (Marchetto et al., 2010; Cheung et al., 2012; Djuric et al., 2015; Chin et al., 2016). The cell lines used in these studies harbored mutations at p.R306C (Cheung et al., 2011; Chin et al., 2016), p.L386R (Marchetto et al., 2010), and p.T158M (Cheung et al., 2011), and deletions at p.G16Efs^∗^22, g.61340_67032delinsAGTTGTGCCAC (p.?) and g.67072_67200del (p.?) (Djuric et al., 2015). Consistent with post-mortem and animal studies, all RTT iPSC cell lines have shown a significant decrease in cell soma size, with a 14.31-fold ± 4.38% decrease (Marchetto et al., 2010) and a 20–25% reduction (Chin et al., 2016) compared to controls. Post-mortem and mature murine neurons show fewer synapses and reduced dendritic branching (Belichenko et al., 2009; Marchetto et al., 2010; Baj et al., 2014), which has been corroborated by recent RTT-iPSC differentiated neuron studies in both female (Chapleau et al., 2009) and male (Yoo et al., 2017) cell lines. Both RTT-iPSC-derived neurons from a female cell line with a point mutation (p.^∗^487Trpext^∗^27) and a female cell line with a frameshift mutation that results in a truncated transcript (p.V247^∗^) showed significantly reduced dendritic branching. Interestingly, a reduction in expression of the tumor suppressor protein p53 was also observed (Ohashi et al., 2018). p53 is known for its involvement in cellular senescence and activates downstream pathways of DNA repair and apoptosis. Ohashi et al. (2018) found that upon inhibition of p53, dendritic branching in RTT-iPSC neurons was restored, suggesting a role for p53 in dendritic branching. This finding is consistent with previous murine studies, where induced p53 expression led to defective dendritic branching in RTT (Ferrón et al., 2009).

Interestingly, neuronal morphological traits have been inconsistent amongst RTT iPSC studies. For instance, two male cell lines harboring mutations at p.Q83^∗^ and p.N126I showed no difference in NPC morphology compared to their respective paternal control (Kim et al., 2011). In contrast, another study reported that the average neurite length was significantly higher in controls than in mutants (Yoo et al., 2017). It was also suggested that there might be a link between the neuronal cell adhesion molecule L1 and MeCP2 activity (Muotri et al., 2010; Yoo et al., 2017). L1 plays crucial roles in multiple cellular functions, including neuronal migration, adhesion, differentiation and survival, as well as neurite outgrowth, axonal targeting, myelination, synapse formation and synaptic plasticity (Yoo et al., 2017) and thus may be a therapeutic target for RTT. Indeed, upon measuring L1 protein expression, a 2.6-fold decrease in expression was observed in RTT-NPCs compared to wild type-NPCs. Phenotypic rescue was observed when cells were transfected with an L1 plasmid construct, suggesting that L1 may be a key driver in cortical development (Yoo et al., 2017). Reduced maturation was also found in RTT-iPSC-derived neurons, this was shown by decreased expression of the mature neuronal markers Tuj (βIII tubulin) (Kim et al., 2011) and MAP2 (Ohashi et al., 2018) in RTT-iPSC neurons compared to controls.

#### Modeling the Synaptic and Electrophysiological Phenotype of RTT Cells

Rett syndrome is known to be a disorder of neuronal plasticity (Dani et al., 2005; Zhang et al., 2008; Maliszewska-Cyna et al., 2010) where aberrant expression of receptors and neurotransmitters suggest that MeCP2 is required to maintain synaptic excitation and inhibition that are fundamental to normal circuitry function (Blackman et al., 2012). This is supported by evidence that RTT patients display elevated glutamate levels and reduced GABA levels (Livide et al., 2015).

Synaptic function has been measured in neurons derived from RTT-iPSCs harboring different mutations. Comparison of the excitatory synapse number between RTT neurons (p.R306C, p.L386Rfs^∗^, p.Q244X, p.T158M) and control iPSC-derived neurons has demonstrated a significant reduction in synapse number (measured according to VGLUT1 puncta), pointing to glutamate transport defects in RTT iPSC differentiated neurons (Marchetto et al., 2010). These electrophysiological defects have been validated in neurons from iPSCs with different mutations by Chin et al. (2016) and Djuric et al. (2015). Despite the use of different measurement techniques, synaptic transmission in female iPSC-derived neurons (p.R306C and p.L386Rfs) (Chin et al., 2016) and g.61340_ 67032delinsAGTTGTGCCAC (p.?) and g.67072_67200del (p.?) (Djuric et al., 2015) has shown significantly lower postsynaptic frequency in RTT-iPSCs compared to the controls, supporting a *MECP2*-dependent synaptic dysfunction phenotype.

Delayed GABA functional switching from excitatory to inhibitory, mediated by the membrane potassium chloride co-transporter KCC2, has been demonstrated in male RTT-iPSCs (p.Q83^∗^) (Tang et al., 2016). In addition, RNA-seq profiling revealed a disruption of GABAergic circuits in RTT-iPSC-derived neurons harboring the p.T158M and p.R306C mutations (Landucci et al., 2018). Functional RTT studies using whole-cell patch clamping revealed reduced spontaneous GABAergic currents and amplitude as well as hyper-excitability in the RTT-iPSC neurons, which is in line with previous mouse model studies (Marchetto et al., 2010; Chin et al., 2016).

Synaptic function and plasticity have been studied in RTT derived iPSCs and controls using several methods including electrophysiology, gene expression and imaging. However, each method has its own challenges. Whole cell electrophysiological recordings provide high temporal and spatial localization, but the restriction of measurements to individual neuronal cells limits its efficacy in understanding complex network interactions (Glasgow et al., 2019). On the other hand, techniques including immunohistochemistry or transcriptomic analysis provide high neuroanatomical specificity and can allow for quantitative comparison, although they exhibit low temporal acuity. It is known that synaptic transmission is a complex phenomenon, but recent developments of more accurate techniques to measure plasticity, such as the use of optical tools alongside classical electrophysiological techniques, are providing new insights into the traditional interpretation of synaptic function. Moving forward, multiple experimental techniques should be used, when possible, to evaluate all possible sites of action.

#### Modeling Therapeutic Targets in RTT Cells

Currently, there are two major targets for therapy for RTT syndrome: therapeutic targets downstream of MeCP2 and gene therapy strategies. The iPSC model provides unique opportunities both for gene therapy and for drug screening, where variant-specific biochemical activities can be used as readouts of drug action. Given the reversibility of the RTT phenotype in mice (Guy et al., 2007), the possibility of rescuing the phenotype *in vitro* by treatment with candidate drugs has been an obvious area of exploration (Marchetto et al., 2010). The read-through drug gentamicin has been tested on iPSCs harboring the nonsense p.Q244^∗^ mutation to determine whether a read-through of the premature stop codon could be elicited (Marchetto et al., 2010). Treatment with gentamicin resulted in increased MeCP2 protein levels and a concomitant number of glutamatergic synapses in iPSC-derived neurons in culture (Marchetto et al., 2010).

A deficiency in the expression of potassium chloride cotransporter 2 (KCC2) in RTT iPSC-derived neurons has been demonstrated to result in a delayed GABA functional switch from excitation to inhibition (Tang et al., 2016). MeCP2 regulates KCC2 through REST, hence controlling GABA functions in neurons. Recent studies have shown that inhibiting NKCC1 (chloride transporter with opposite functions to KCC2) can be used to treat autism and fragile X syndrome (Tang et al., 2016). Given the critical role of IGF1 in neurodevelopment and its capacity to rescue glutamatergic deficits, the effects of insulin like growth factor (IGF1) were tested in neuronally differentiated RTT-iPSCs. Although MeCP2 expression did not change, KCC2 was increased significantly, suggesting that IGF1 may upregulate KCC2 independently of MeCP2 activity (Tang et al., 2016). The effects of IGF1 treatment have been investigated by another group (de Souza et al., 2017) in RTT (p.Q83^∗^) and control iPSCs (HUES6) by measuring the global gene expression profiles of RTT-iPSCs and control lines at different stages of differentiation. This *in vitro* study showed that the thyroid hormone receptor alpha 3 gene (TRalpha3), which encodes a hormone receptor critical for brain development, displayed a different expression profile in the mutant cells. It was demonstrated that treatment with IGF1, mediated by TRalpha3, was able to enhance neurite growth in MeCP2-deficient cells, suggesting IGF1 as a treatment option (de Souza et al., 2017, 2019).

Motor and cognitive impairments in RTT mouse models may be related to abnormalities in the cholinergic system (Nag and Berger-Sweeney, 2007) and thus RTT-iPSC-derived neurons have been used to test whether choline could rescue RTT-associated defects. From these studies, choline supplementation was shown to alleviate the synaptic defects in RTT-iPSC-derived neurons in culture (Chin et al., 2016). The action of histone deacetylase 6 (HDAC6) inhibitors has also been tested in RTT-iPSCs. HDAC6 is a cytoplasmic microtubule-associated deacetylase that catalyzes the deacetylation of alpha-tubulin. Disruptions in acetylated α-tubulin and over-expression of HDAC6 have been reported in murine models and RTT fibroblasts (Gold et al., 2015). Recently, the administration of multiple HDAC6 inhibitors (for instance, ACY-1215) was shown to induce a marked increase in acetylated α-tubulin in RTT iPSC-derived neurons (Landucci et al., 2018). These studies demonstrate the capability of iPSC models to recapitulate the molecular abnormalities of RTT and the utility of HDAC6 inhibitors for RTT therapy.

The development of therapeutic strategies like the ones listed in this review (Table 2) needs to take into account the cell type, time of dosing and target; iPSCs demonstrate to be a useful tool to do preliminary studies on treatment strategies. In addition, other therapeutic targets currently being tested include: BRD4 targeting (Xiang et al., 2020); reactivation of the inactive X chromosome [Reviewed in Good et al. (2021)]; and other histone deacetylase inhibitors (including HDAC3) (Nott et al., 2016).

**TABLE 2:** Summary of therapeutic targets and outcomes.

**Study**	**Therapeutic targets**	**Outcome**
Marchetto et al., 2010	Gentamicin	Increased levels of MeCP2 and increased number of glutamatergic synapsis in neuron cultures.
de Souza et al., 2017	IGF1	Showed possible link between IGF1/IGF1R and TRalpha3, overexpression of IGF1R caused neurite improvement and RTT derived neurons
Tang et al., 2016	KCC2	Restoration of KCC2 levels rescues GABA functional deficits in RTT neurons
Chin et al., 2016	Choline	Choline supplementation was shown to alleviate the synaptic defects in RTT-iPSC-derived neurons in culture
Landucci et al., 2018	HDAC6 inhibitor	Increase in acetylated α-tubulin in RTT derived neurons, improving synaptic function.

### Modeling RTT Glial Cells

Glia cells, including astrocytes and microglia, play a critical role in mediating dendritic growth, synapse formation, synaptic function and immune responses in the neuropathology of RTT (Lioy et al., 2011). Despite being expressed at lower levels in astrocytes than in neuronal cells, MeCP2 is critical for astrocyte differentiation and function where MeCP2-deficient astrocytes have abnormal BDNF regulation, cytokine production, and neuronal dendritic formation and maturation (Maezawa et al., 2009). Several RTT studies have demonstrated that astrocytes negatively impact neurons in a non-cell autonomous manner (Ballas et al., 2009; Maezawa et al., 2009; Lioy et al., 2011), revealing distinct roles for astrocytes and neurons in disease progression, where mutant neurons primarily initiate the disease state and mutant astrocytes influence disease progression. Early *in vitro* studies demonstrated that co-cultured mutant mouse astrocytes, and the supplementation of mutant astrocyte conditioned medium, failed to support normal dendritic morphology of wild type and mutant hippocampal neurons (Ballas et al., 2009). Further *in vivo* studies show that re-expression of MeCP2 in astrocytes reverted RTT-specific phenotypic behaviors. Re-expression also restored aberrant dendritic morphology and increased the levels of the excitatory glutamate transporter VGLUT1 in a non-cell autonomous manner (Lioy et al., 2011).

More recently, RTT patient-derived iPSCs have been successfully differentiated into glial cells including astrocytes and microglia in three different patient cell lines (p.V247^∗^, p.R294^∗^, and p.R306C) (Williams et al., 2014). To understand whether human MeCP2-deficient astrocytes have a non-cell-autonomous effect on mouse neurons, RTT patient iPSC-derived astrocytes differentiated from late-phase isogenic astroglia progenitors were co-cultured with either wild type or mutant murine P0 primary hippocampal neurons. In agreement with previous studies, the mutant astrocytes adversely affected the morphology and function of the murine neurons in a non-cell-autonomous manner (Williams et al., 2014). Neuronal deficits caused by mutant RTT astrocytes were partially rescued by introduction of insulin-like growth factor 1 (IGF-1) and its truncated derivative glycine-proline-glutamate (GPE) (Williams et al., 2014). Short-term treatment with IGF-1 or GPE in co-culture combinations of mutant and wild type interneuron/astrocyte demonstrated an indirect effect of IGF-1 treatment on neurons with a direct impact on astrocytes (Williams et al., 2014).

Induced pluripotent stem cell studies generated from female monozygotic twins harboring a frameshift mutation (p.G269Afs^∗^) in *MECP2* revealed abnormal growth patterns and gene expression in differentiated mutant astrocyte cells compared to their isogenic controls. In particular, the glial marker GFAP (glial fibrillary acidic protein) was shown to be dysregulated in MeCP2-deficient neural cells, suggesting that abnormal astrocytic differentiation is involved in the pathogenesis of disease and a likely therapeutic target for RTT (Andoh-Noda et al., 2015).

Astrocytes have also been generated from male RTT-iPSC lines harboring loss of function mutations (p.Q83^∗^, p.N126I). In comparison to iPSC lines from unaffected fathers (WT83, WT126), mutant lines showed significantly reduced levels of expression of astrocytic markers (Kim et al., 2019), suggesting that RTT-NPCs can differentiate into GFAP-positive glia, albeit at a reduced capacity. In this study, proteomic analyses of molecular pathways identified abnormal upregulation of LIN28, a driver of neural differentiation essential for cell-fate regulation and developmental timing in *MECP2* mutant-expressing NPCs compared to controls. From these studies, LIN28 expression was found to be inversely correlated with GFAP-positive glia generation, indicating that high LIN28 expression in mutant NPCs hampers astrocytic differentiation by reducing neuronal synapse density (Kim et al., 2019). Knockdown of LIN28 allowed for a partial reversal of synaptic deficiencies in MECP2 mutant lines (Kim et al., 2019).

It has been shown that restoration of MeCP2 in neurons alone was not able to recover the phenotype in mouse brains, suggesting the involvement of other CNS cell types such as glia (Alvarez-Saavedra et al., 2007). The above-mentioned studies have successfully used iPSC-derived astrocytes and microglia to investigate the effects of these cell types in RTT pathophysiology, but the exploration of potential therapeutic candidates targeting glia remains largely unexplored and continued investigation is needed to understand the involvement of glia in RTT.

### Modeling the Impact of X-Chromosome Inactivation

X-chromosome inactivation (XCI) is a phenomenon of female (XX) somatic cells, where one X chromosome undergoes inactivation in a stochastic manner such that genes of only one X-chromosome are expressed in each cell, thus balancing the dosage of X-linked genes with the male (XY) cells. This is critical to RTT as *MECP2* is located on the X chromosome and subjected to random silencing that can result in mosaic expression of *MECP2* with some cells expressing the mutant *MECP2* allele and others the wild type allele.

Female iPSCs have been shown to retain the inactive X-chromosome (Tchieu et al., 2010), in contrast to murine pluripotent stem cells, which can express both X-chromosomes (Maherali et al., 2007). However, it has been demonstrated that despite the X chromosome status at the iPSC stage, iPSCs that have two active X chromosomes will inactivate an X chromosome upon neuronal differentiation (Marchetto et al., 2010), thus regaining the XCI status found in somatic cells.

The activation status of the X chromosome is critical when using iPSCs as cellular models for RTT research and thus warrants careful investigation. The XCI status has been studied in RTT iPSCs by several groups (Marchetto et al., 2010; Amenduni et al., 2011; Ananiev et al., 2011; Cheung et al., 2011; Kim et al., 2011; Pomp et al., 2011). Interestingly, the XCI status of the different lines and clones has been inconsistent (Table 1). Some groups observed clones that reactivate the previously inactive X chromosome and in some cases up to 95% of the cells in the line (Marchetto et al., 2010). In contrast, a number of groups observed retention of the inactive X chromosome (Amenduni et al., 2011; Ananiev et al., 2011; Cheung et al., 2011; Pomp et al., 2011), reactivation of the inactive X (Marchetto et al., 2010) or combinations of both (Kim et al., 2011). These findings suggest that during reprogramming, some clones may erase the memory of the XCI status. This erosion of the inactive X chromosome characterized by loss of XIST coating or DNA methylation, has been observed in many X-linked iPSC studies (Luo et al., 2014; Geens et al., 2016; Vallot et al., 2017). Although this phenomenon is not completely understood, it is speculated to be an artifact of culture conditions. Derivation and cell culture conditions have been shown to have a significant impact on the XCI status, and include oxygen percentage and supplementation with small molecules like sodium butyrate and 3-deazaneplanocin A or antioxidants (Lengner et al., 2010). Obviously, the use of male iPSC would avoid the issue of XCI, however, as most Rett patients are female, this is not relevant for most cell lines. Clearly the optimization of culture methods is warranted in order to obtain the natural XCI state of the iPSC line.

There is much speculation regarding the cause of the differences in the XCI status of these iPSC cell lines. XCI status may be influenced by physiological stress, as in hESCs where changes in CO_2_ levels result in the expression of both alleles of the X-linked gene (Lengner et al., 2010). However, iPSCs can maintain their XCI status through many culture passages and freeze-thaw events (Kim et al., 2011). Despite the origin of each cell line, the culturing conditions of the lines and the reprogramming methods used have been broadly similar, although subtle variations in reprogramming protocols cannot be completely ruled out. Indeed, while similar programming methods were used to generate the iPSCs (Ananiev et al., 2011; Cheung et al., 2011; Kim et al., 2011; Pomp et al., 2011), reactivation of the previously inactive X indicates that the stability of the XCI status is not always assured and other unknown factors may be at play. Thus, the programming methods may affect the XCI status of the iPSCs and should be carefully considered when using these cell lines. Regular assessment of the XCI status, in particular the skewed pattern, should be undertaken during the differentiation process to validate the XCI status of the cell line.

As observed in RTT patients, skewed XCI has also been reported in neuronally differentiated iPSCs (p.R294^∗^, p.T158M, and p.V247^∗^) (Ananiev et al., 2011). A drastic skewing was found in iPSC-derived neurons harboring a p.L386R*fs*^∗^ mutation (6:4 to 98:2), despite the originating fibroblasts showing equal expression of both alleles (55:45) (Marchetto et al., 2010). Extreme XCI skewing was also found in the Δ3–4 iPSC (96:4 to 99:1), p.T158M iPSC (91:9 to 99:1) and p.R306C iPSC (84:16 to 81:19) lines, with the originating fibroblasts exhibiting a balanced pattern of XCI. In addition, some clones from the Δ3–4 iPSC line were skewed toward the same parental X chromosome, and others toward the other parental X chromosome, whereas the skewed pattern in the p.T158M and p.R306C lines retained the inactive parental chromosome. This demonstrates that inactivation of the X chromosome in iPSCs derived from the fibroblasts of these patients seems to be non-random. Despite this, the XCI pattern of *MECP2* is retained during neuronal differentiation, allowing the generation of isogenic controls (expressing wild type *MECP2*) and experimental iPSC lines (expressing mutant *MECP2*). Recommendations to ensure that the iPSCs and derived cells retain the XCI status of the parental patient cell lines, whether they originate from fibroblasts, urine or blood, require validation by mRNA expression of which X chromosome is being expressed, and selection of iPSC clones that only express the same X chromosome and skewing as the parental line.

## Modeling RTT Using Brain Organoids

Despite their simplicity and utility, 2D cell cultures do not completely recapitulate the complex functional interaction between different cell types and the 3D dimensional architecture of neural tissue in the brain. Capitalizing on the capacity of iPSC-derived neural cells to differentiate in response to mechanical and chemical stimuli, and the ability of stem-cell derivatives to self-organize into organotypic structures that resemble the brain, enables the production of 3D organoids *in vitro*. The discovery that neural rosettes (neural progenitors) can be generated from embryonic stem cells has powered the development of cerebral organoids (Lancaster et al., 2013; Lancaster and Knoblich, 2014). Subsequent culture methods facilitated “self-patterning” of cells (Lancaster et al., 2013) and “direct patterning” toward defined brain regions such as the cortex, forebrain (Qian et al., 2016) and midbrain (Paşca, 2019). Single-cell RNA-sequencing analysis has shown that whole-brain organoids generated by “self-patterning” display a high degree of variability and low reproducibility of cell types in comparison to “direct patterning” (Velasco et al., 2019). Direct patterning protocols use morphogenetic factors and inhibitors to drive the patterning of specific brain regions to allow complex assembly of different brain regions (the assembloid) as well as the inclusion of other cell types such as microglia, endothelial cells and even brain blood barrier-like structures. Brain organoid models have facilitated investigations into the intricate cellular interaction during neurodevelopment and the disease process, providing a human-like model for screening therapeutics and preclinical testing of potential therapies. Different human brain organoid culturing methods have been reviewed in more detail elsewhere (Wang, 2018).

Two recent studies using RTT patient iPSC-derived brain organoids have characterized the morphology and synaptic function of mutant neurons (Trujillo et al., 2018) and the dysregulation of signaling pathways (Mellios et al., 2018). Despite differences between the two methods, their impacts on the field have been significant. Trujillo et al. (2018) developed cortical brain organoids by culturing male patient-derived iPSCs (p.Q83^∗^ and p.N126I) and the paternal controls in the presence of dual SMAD inhibition and Y-27 (ROCK inhibitor) in a spinning orbital shaker. To enhance the growth of the organoids and initiate neuroepithelium differentiation, the culture was initially supplemented with fibroblast growth factor (FGF), epidermal growth factor (EGF) and then BDNF, GDNF, NT-3, L-ascorbic acid, and dibutyryl cAMP. Organoids were then matured in an orbital shaker for 10 months (Trujillo et al., 2018). In contrast, Mellios et al. (2018) generated spheroids from two female RTT patient cell lines carrying a missense mutation (p.R106W) and a frameshift deletion (p.E235fs), respectively, and the corresponding isogenic controls, and two wild type control lines (male GM08330 and female GM23279). Spheroids were made by generating embryo bodies in ultra-low attachment well plates with neural induction media, using iPSCs previously grown on mouse embryonic cells (MEFs). Spheroid differentiation occurred in B-27 supplemented media followed by dual SMAD inhibition (SB-431542 and LDN-193189) at the induction phase. The spheroids were embedded in Matrigel for maturation in a bioreactor for 5 weeks (Mellios et al., 2018).

Mellios et al. (2018) explored the role of miRNAs in neurogenesis by electroporating the organoids with a MeCP2 short hairpin RNA (shRNA) construct. Novel MeCP2-regulated miRNAs were identified, such as miR-199 and miR-214 that can differentially regulate the extracellular signal-regulated kinase (EKR/MAPK) and protein kinase B (PKB/AKT) signaling pathways. By inhibiting miR-199 and miR-214 expression at the neural progenitor stage, AKT and ERK activation was restored, leading to amelioration of the RTT-like phenotype (Mellios et al., 2018). Trujillo et al. (2018) used the organoid models to investigate the electrophysiological activity of RTT neurons. To deduce whether the organoids could recapitulate the synaptic function of the human brain, synaptic maturation was measured using microelectrode arrays (MEAs) and compared to electroencephalogram data of preterm infants by machine learning. Neurons in the *MECP2*-deficient organoids showed significant reductions in cell size, neural protrusions, spine-like density and synaptic puncta. In addition, reduced neural activity indicated an absence of network oscillations, which was not found in the isogenic controls at the same age *in vitro*. Not only were significant differences in neuronal morphology and function observed between RTT and wild type organoids, but this work also provides a cortical organoid model that recapitulates the synaptic function of the *in vivo* prenatal brain, the incremental emergence of glial cells, and the formation of inhibitory neurons by 6 months in culture (Trujillo et al., 2018). As RTT post-mortem brain tissue is not easily accessible, 3D organoid models provide an exciting avenue to explore disease mechanisms and potential therapies.

Human brain organoids offer the opportunity to better understand neural circuits, enabling elucidation of the mechanisms that underlie the properties of human circuits. However, despite considerable progress in the field, there has been little evidence to demonstrate the establishment of reliable cortical circuits. One of the obvious obstacles is that brain organoids lack critical cell types of the human cortex, including endothelial cells, and microglia and oligodendrocytes. Recently, efforts have been made to implement vascular structure in human brain organoids by adjusting cultivation protocols, introducing microfluidic devices, and transplanting organoids into immunodeficient mice (Cakir et al., 2019; Shi et al., 2020). Another confounding factor is that even though organoids show some structural features of cortical layering and ventricular zones, more complex levels of cortical organization such as gyrification, radial glial scaffolds and neuronal connections are still to be established. Finally, an overriding issue regarding brain organoids as a model is their relative immaturity. Whether more complex structures can be developed that possess improved organization and model later stages of development, remains to be seen.

### Limitations of the Brain Organoid Model

One caveat in the utility of iPSCs is related to their variability in lineage propensity and this has a confounding effect on the consistency and reproducibility of the cellular composition of the organoid. iPSC derivation and differentiation is a multi-step processes and experimental procedure variations can produce different outcomes (Popp et al., 2018). Often, the impact of variation can overpower any biological differences, especially where sample sizes are small (Ghaffari et al., 2018). Studies have also confirmed that the individual donor’s genetic background and differences in differentiation protocols might significantly influence the methylation landscape, affecting pluripotency between iPSCs from different donors (de Boni et al., 2018). Analysis has shown that induction and differentiation can also introduce variation themselves (Schwartzentruber et al., 2018). Hence, measures need to be put in place to minimize variation while differentiating iPSCs, such as consistencies in passage number and differentiation protocols, including culture media and matrices.

Another known and reported limitation is the inadequate functionality of the tissue in the organoid such as the lack of vascularization. Vascularization supports efficient transport of oxygen and nutrients to the core of the brain organoids, which are critical for proper growth and differentiation, because non-vascularized organoids often display tissue necrosis. An appealing approach is the xenograft transplantation of brain organoids into mice, allowing the murine blood vessels to transport oxygen and nutrients to support the growth of the transplanted organoids (Mansour et al., 2018). Concurrently a variety of *in vitro* models for vascularization are also being devised including endothelial cells cultured on microfluidic channels made with degradable materials to resemble the blood vessels in tissues (Bhatia and Ingber, 2014; van Duinen et al., 2015).

The most critical limitation is related to the ability to mimic the normal development trajectory of the human brain. iPSC-derived patient neurons and brain organoids are imperfect in that they are highly heterogenous, only replicate a specific brain region, and are not connected to the peripheral nervous system, amongst other shortcomings. Directed patterned and self-patterned models have been developed to replicate the anatomically relevant spatial organization found *in vivo.* A single cell RNA sequencing study has shown that directed patterned organoids have a higher rate of reproducibility over self-patterned whole brain organoids (Velasco et al., 2019). However, the former approach only allows for specific regions of the brain to be generated including cerebral cortex, hippocampus (Sakaguchi et al., 2015) midbrain (Jo et al., 2016; Qian et al., 2016) forebrain (Velasco et al., 2019) and cerebellum (Muguruma et al., 2015) with no studies yet having combined these regions, nor has the establishment of functional neuronal connectivity or neuron-glia interactions been demonstrated.

## Challenges and Future Directions

Currently, brain organoids do not reproduce the requisite tissue complexity of the human brain, and models have yet to recapitulate the developmental stages of the brain of a young child for studying the postnatal manifestation of neurodevelopmental disorders. RTT patients develop normally from 6 to 18 months after birth, which is followed by a developmental trajectory regression where they lose their ability to control their hand movements, they stop walking and talking, and they develop phenotypes such as microcephaly, seizures, autism, prolonged QT intervals, and breathing problems including apneas. Symptoms range in severity with limited phenotype-genotype correlations. The expression of *MECP2* is tightly regulated both temporally during development and spatially throughout the brain. As *MECP2* is an X-linked disorder, it is subjected to X-chromosome inactivation, resulting in the skewing of either the wildtype or mutant allele. All these unusual characteristics make it very challenging to study the pathophysiology of disease in *in vitro* cellular expression systems and in mouse models as they do not completely recapitulate the disease that is observed in human subjects.

Rett syndrome patient iPSC-derived neurons and brain organoids are effective models to address several specific phenotypes such as synaptic dysfunction, glutamatergic neuron morphology, electrophysiological and synaptic dysfunction, GABA-ergic dysregulation, non-cell autonomous effects of astrocytes, transcriptional dysregulation, neurogenesis, and miRNA target sites. In addition to this, therapeutic molecules have also been tested, broadening our understanding of iPSC-derived models as well as the pathophysiology of RTT. However, one of the critical questions in the field is how developmental delay and regression can be interrogated using these models, and whether they can inform us about how to reverse RTT in humans. The contributions by Guy et al. (2007), demonstrating phenotypic reversal in mice, provided much hope to the RTT community that the disorder was indeed reversable, albeit in murine models. 3D brain organoids that more closely resemble the human brain than the widely used mouse models, may be able to address this question. Cortical organoid studies have recapitulated the features of cortical development including temporal neurogenesis and functional cortical neurons, as well as the appearance of spontaneous glial cell populations (Kadoshima et al., 2013; Qian et al., 2016; Paşca, 2019; Xiang et al., 2020). Further, forebrain organoid development has allowed for the modeling of tangential migration of interneurons into the cortex by fusing dorsal forebrain organoids with ventral forebrain organoids (Kim et al., 2019; Xiang et al., 2020), which has enabled the study of human-specific features of brain development. Studies such as these that address the development of the brain in RTT-iPSC-derived neurons and organoids, together with the refinement of brain organoid technologies, will overcome the existing shortcomings and provide more insight into the developmental delay associated with MeCP2 deficiency. The organoid model has untapped potential for developing personalized therapeutic strategies for RTT and other monogenic neurodevelopmental disorders.

Both the cellular model and the organoid model have their specific utility and limitations. There may be no ideal models, but rather appropriate models that fulfill the requirements of the research question. This review outlines the major phenotypes that have been modeled in RTT-iPSC-derived cells and organoids and their application for drug screening. Impending optimization in modeling technology will enable the creation of high-fidelity disease models for the human brain, which in addition to modeling pediatric neurodevelopmental disorders, will allow the characterization of normal and abnormal neurogenic differentiation and tracking of the disease trajectory through pre and postnatal development.

## Author Contributions

FH wrote the manuscript. BC summarized studies used in the review. LR checked the genetic nomenclature. LCC, LR, PPLT, and WAG contributed to the drafts and revisions. PLTT and WAG framed the concept and structure of the review. All authors reviewed and approved the final manuscript.

## Conflict of Interest

The authors declare that the research was conducted in the absence of any commercial or financial relationships that could be construed as a potential conflict of interest.

## Publisher’s Note

All claims expressed in this article are solely those of the authors and do not necessarily represent those of their affiliated organizations, or those of the publisher, the editors and the reviewers. Any product that may be evaluated in this article, or claim that may be made by its manufacturer, is not guaranteed or endorsed by the publisher.
