# Supplementary material for: Pre-clinical Investigation of Rett Syndrome Using Human Stem Cell-Based Disease Models
Source: Front Neurosci. 2021 Aug 25;15:698812. doi: 10.3389/fnins.2021.698812 (PMC8423999; doi:10.3389/fnins.2021.698812)
Supplement: Supplementary file 1 [file Table_1.docx]

**Supplementary Table 1** List of publications reviewed, including location of the mutation, ID and gender as well as control lines used.

| Publication | Synopsis | Cellular model | *MECP2* Mutant Lines used in the study: ID/Mutation/gender | Control lines used in the study |
| --- | --- | --- | --- | --- |
| Ananiev  (Ananiev et al., 2011) | Owing to XCI, isogenic pairs from wild type and mutant iPSCs were developed and phenotypic studies carried out to set up an *in-vitro* model for RTT. | iPSC-neurons | 1. GM11270/c.916C>T, p.R306C/female 2. GM17880/c.473C>T, p.T158M/female 3. GM07982/c.705delG, p.V247*/female 4. RS0502/c.916C>T, p.R294*/female | Age Matched female (AG07306) |
| Andoh-Noda  (Andoh-Noda et al., 2015) | iPSC RTT model demonstrates that MeCP2 is relevant in the process of neuronal cell differentiation. RTT-iPSCs derived astrocytes show perturbed gene expression and accelerated astrocyte formation. | iPSC- neurons and astrocytes | 1. RS1/RS2/p.G269Afs, c.806delG)/female | Isogenic controls generated from patient cells |
| Cheung  (Cheung et al., 2011) | Demonstrated inactive X-chromosome in a nonrandom XCI pattern and performed cellular phenotypic analysis demonstrating a reduction in soma size of RTT-iPSC derived neurons. | iPSC-neurons | 1. Δ3–4/ g.61340_67032delinsAGTTGTGCCAC and g.67072_67200del/female 2. GM17880/c.473C>T, p.T158M/female 3. GM11270/c.916C>T, p.R306C/female | 1. Wild type male (CRL-2522) 2. Wild type female (CCL-186) |
| Chin  (Chin et al., 2016) | Demonstrated RTT iPSC-derived cells show reduced soma size, and decreased synaptic input, Supplementation with choline rescued these defects. | iPSC neurons | 1. RTT1/c.916C>T, p.R306C/female 2. RTT2/c.1155del32, p.L386R*fs*/female | Isogenic control generated from RTT2 cells (CTRL) |
| De Souza  (de Souza et al., 2017) | TRalpha3 is differentially expressed in RTT models and may improve neurites growth in RTT-derived neurons. | iPSC- neurons | RTT/p.Q83*/male | WT83 male control |
| De Souza  (de Souza et al., 2019) | TH-related genes, such as deiodinases, are altered in RTT samples when compared. Treatment of neural cells with TH increased MAP2 and synapsin-1 expression. | iPSCs- neurons | Q83X/p.Q83Xfs/male | Wild type male |
| Djuric  (Djuric et al., 2015) | RTT iPSCs-derived neurons showed decreased soma size reduced dendritic complexity and decreased cell capacitance including dysfunction in action potential generation, voltage-gated Na+ currents, and miniature excitatory synaptic current frequency and amplitude. Soma size was rescued by MECP2e1 transduction. | iPSC- neurons | RTTe1 - NM_001110792.1/c.47_57del, p.G16Efs*22/female | Isogenic control generated from patient cells |
| Hinz  (Hinz et al., 2019) | Fibroblast pre-sorting followed by episomal reprogramming can be used to reliably generate iPSC lines with specified X-chromosomal phenotype such as RTT. | iPSC-neurons | 1. RTT-FB DEL (Deletion within exon 3 and 4)/female 2. RTT-FB R255X/c.763C>T, p.R255* 3. RTT-FB R270X/c.808C>T, p.R270* | Isogenic control generated from patient cells |
| Kim  (Kim et al., 2011) | Mutant monoallelic or biallelic RTT-iPSCs displayed a defect in neuronal maturation consistent with RTT phenotype. | iPSC-neurons | 1. RTT1, GM17880/c.473C>T, p.T158M/female 2. RTT2, GM16548/c.730C>T, p.Q244*/female 3. RTT3, GM07982/c.705delG, p.V247*/ female 4. RTT4, GM11270/c.916C>T, p.R306C/ female 5. RTT5, GM17567/c.1461A>G, p.W487*/ female | 1. PGP1-iPS1 2. PGP9f-iPS1 3. 551-iPS-K1 4. hESCs–H1 5. hESCs–EOS2 |
| Kim  (Kim et al., 2019) | Overexpression of LIN28 protein in control NPCs suppressed astrocyte differentiation and reduced neuronal synapse density, whereas downregulation of LIN28 expression in mutant NPCs partially rescued this synaptic deficiency. | iPSC derived neural progenitor cells | 1. Q83X/p.Q83*/male 2. N126I/c.377A>T, p.N126I/male | Non-affected father |
| Landucci  (Landucci et al., 2018) | Profiling by RNA-seq revealed a prominent GABAergic circuit disruption and a perturbation of the cytoskeleton. A significant decrease of acetylated α-tubulin h was reverted by HDAC6i’s. | iPSC-neurons | 1. c.473C>T, p.T158M/female 2. c.916C>T, p.R306C/female | 1. Isogenic control derived from p.Thr158Met patient 2. BJ Human Cell Line 3. SRA01/04 cell line |
| Livide  (Livide et al., 2015) | GRID1 expression is downregulated in both MECP2- and CDKL5-mutated iPSCs and upregulated in neuronal precursors and mature neurons. | iPSC- neural progenitors and neurons | NM_ 004992.3/c.916C>T, p.R306C | BJ control clone |
| Marchetto  (Marchetto et al., 2010) | Neurons derived from RTT-iPSCs showed fewer synapses, reduced spine density, smaller soma size, altered calcium signalling and electrophysiological defects. Data showed early alterations in developing human RTT neurons. Selected compounds rescued synaptic defects. | iPSC- neural progenitors and neurons | 1. GM11270/c.916C>T, p.R306C/female 2. GM11272/c.1155del32, p.L386R*fs*/female 3. GM16548/c.730C>T, p.Q244*/female 4. GM17880/c.473C>T, p.T158M/female | 1. AG09319 2. CRL2529 3. WT-126 4. WT-33 5. WT- ARDC40 |
| Muotri  (Muotri et al., 2010) | RTT NPCs show increased susceptibility for L1 retro transposition. | iPSC- neural progenitor cells and neurons | - RTT/GM11272/c.1155del32, p.L386R*fs*/female | 1. AG09319 2. hESCs-Cyt25 |
| Nguyen  (Nguyen et al., 2018) | Electrical stimulation increased cell maturation and improvements in cell morphology of the RTT cells | iPSC- neurons | RTT/c.1155del32, p.L386R*fs*/female | 1. Isogenic control from patient cells |
| Ohashi  (Ohashi et al., 2018) | KO MECP2 cell line showed signs of stress, including induction of P53, and senescence. P53 induction may affect dendritic branching | iPSC- neurons | 1. GM17567/p.*487Trpext*27, c.1461A>G/female   GM07982/c.705delG, p.V247*/female | Isogenic control from patient cells |
| Pomp  (Pomp et al., 2011) | iPSC colonies contain an inactive X, and all colonies made from the same donor fibroblasts contain the same inactive X chromosome. "Skewing" towards a particular dominant, active X seen in reprogramming and fibroblast culture can be alleviated by overexpression of telomerase. | iPSC- neurons | 1. GM17880/c.473C>T, p.T158M/female 2. GM11272/c.1155del32, p.L386R*fs*/female | 1. WI-38 (GM06814)   Non-affected control donor |
| Tang  (Tang et al., 2016) | Restoration of KCC2 levels rescues GABA functional deficits in RTT iPSC derived neurons, showed that MeCP2 regulated KCC2 expression by inhibiting RE1-silencing transcriptional factor. | iPSC- neurons | 1. Q83*/p.Q83*/male | 1. Non-Affected Father cell line. 2. WT-126 3. WT-33 |
| Williams  (Williams et al., 2014) | Morphology and function of wild type neurons are affected when co-cultured with RTT astrocytes affect, IGF1 and GPE can partially rescue neuronal deficits caused by RTT astrocytes. | iPSC- astrocytes co-cultured with WT neurons | 1. GM11270/c.916C>T,p.R306C/female 2. GM07982/c.705del, p.V247X/female   RS0502/c.916C>T, p.R294X/female | 1. Isogenic control from patient cells |
| Yoo  (Yoo et al., 2017) | Decreased expression of L1 and neuritogenesis was shown in RTT NPCs Positive correlations between MeCP2 and L1, and normalisation of cell survival was observed. Expression of L1 in RTT NPCs enhanced neuritogenesis and soma size. | iPSC- neurons and neural progenitor cells | 1. Q83*/p.Q83*/male | Non-affected father |
| Zhang  (Zhang et al., 2016) | Soft 3D systems mimic the brain environment and accelerates maturation of iPSC and derived NPCs, yielding electrophysiological active neurons within just 3 weeks. | iPSC- neurons and neural progenitor cells | 1. RTT/p.Q83*/male   RTT/c.377A>T, p.N126I/male | Non-affected father |
| Mellios  (Mellios et al., 2018) | MeCP2 short hairpin RNA knockdown was utilized to identify novel MeCP2-regulated miRNAs enriched during early human neuronal development miR-199 and miR-214 were increased during early brain development and to differentially regulate extracellular signal-regulated kinase | Brain spheroids | 1. RTT GM11273/c.316C > T, p.R106W/female 2. RTT GM07982/c.705delG, p.E235fs/female | 1. WT male GM08330   WT female GM23279 |
| Trujillo  (Trujillo et al., 2018) | Human cortical organoids that dynamically change cellular populations during maturation and exhibited consistent increases in electrical activity over the span of several months were developed. The oscillatory activity transitioned to more spatiotemporally irregular patterns, and synchronous network events resembled features like those observed in preterm human electroencephalography. | Cortical organoids | 1. RTT/p.Q83*/ male 2. RTT/c.377A>T, p.N126I/ male | 1. Non-affected paternal cell lines. |
|  |  |  |  |  |
